# Differential neuronal vulnerability identifies IGF-2 as a protective factor in ALS

**DOI:** 10.1038/srep25960

**Published:** 2016-05-16

**Authors:** Ilary Allodi, Laura Comley, Susanne Nichterwitz, Monica Nizzardo, Chiara Simone, Julio Aguila Benitez, Ming Cao, Stefania Corti, Eva Hedlund

**Affiliations:** 1 Department of Neuroscience, Karolinska Institutet, Retzius v. 8, 171 77 Stockholm, Sweden; 2 Dino Ferrari Center, Neuroscience Section, Department of Pathophysiology and Transplantation, University of Milan, Neurology Unit, Istituto Di Ricovero e Cura a Carattere Scientifico Foundation Ca’ Granda Ospedale Maggiore Policlinico, Milan 20122, Italy

## Abstract

The fatal disease amyotrophic lateral sclerosis (ALS) is characterized by the loss of somatic motor neurons leading to muscle wasting and paralysis. However, motor neurons in the oculomotor nucleus, controlling eye movement, are for unknown reasons spared. We found that insulin-like growth factor 2 (IGF-2) was maintained in oculomotor neurons in ALS and thus could play a role in oculomotor resistance in this disease. We also showed that IGF-1 receptor (IGF-1R), which mediates survival pathways upon IGF binding, was highly expressed in oculomotor neurons and on extraocular muscle endplate. The addition of IGF-2 induced Akt phosphorylation, glycogen synthase kinase-3β phosphorylation and β-catenin levels while protecting ALS patient motor neurons. IGF-2 also rescued motor neurons derived from spinal muscular atrophy (SMA) patients from degeneration. Finally, AAV9::*IGF*-*2* delivery to muscles of SOD1^G93A^ ALS mice extended life-span by 10%, while preserving motor neurons and inducing motor axon regeneration. Thus, our studies demonstrate that oculomotor-specific expression can be utilized to identify candidates that protect vulnerable motor neurons from degeneration.

Amyotrophic lateral sclerosis (ALS) is a fatal disease characterized by a progressive loss of somatic motor neurons, muscle wasting and paralysis. ALS appears mostly sporadic (sALS), but can be inherited (fALS) due to mutations in e.g. superoxide dismutase 1 (SOD1), TAR DNA-binding protein 43 (TDP-43), Fused in Sarcoma (FUS) and C9ORF72 (chromosome 9 open reading frame 72)^1^,^2^. Importantly, data from fALS models indicate that motor neuron intrinsic factors are crucial for initiation and early progression of degeneration^3^,^4^,^5^. While ALS is characterized by motor neuron loss, certain motor neuron groups are for unknown reasons relatively resistant to degeneration. Among the most resistant are oculomotor neurons^6^,^7^,^8^,^9^, which are located in the brain stem and control eye movement. Consequently, eye-tracking devices are used to enable paralyzed ALS patients to communicate through computers^10^. Thus, an investigation of factors intrinsic to oculomotor neurons in health and disease could reveal mechanisms of neuronal resistance and be the basis for future therapeutic strategies to protect vulnerable motor neurons from degeneration. Towards this goal, we and others have previously shown that resistant oculomotor motor neurons display a distinct mRNA and protein signature compared to other vulnerable motor neuron groups^11^,^12^,^13^. We identified insulin-like growth factor 1 (IGF-1) and 2 (IGF-2) as preferential to oculomotor neurons in the normal rat^11^. This finding is highly compelling in terms of intrinsic neuronal resistance as IGFs are known motor neuron survival factors^14^,^15^. In fact, viral delivery of IGF-1 to motor neurons in the SOD1^G93A^ fALS mouse was neuroprotective and increased the life-span of the mice^16^. The possible motor neuron protective properties of IGF-2 in ALS has not been previously investigated. IGF-2 can bind to IGF-1 receptors (IGF-1R), IGF-2 receptors (IGF-2R) and insulin receptors. IGF-2R has the highest affinity for IGF-2, but the biological effects of IGF-2 are mediated through IGF-1R and/or insulin receptor, just as for IGF-1. The binding of IGF-2 to IGF-1R, which is a receptor tyrosine kinase, leads to activation of PI3K/Akt and survival pathways or activation of mitogen activated protein kinase (MAPK) pathway and proliferation. Insulin receptor activation leads to proliferation. IGF-2 binding to IGF-2R, which lack the intracellular tyrosine binding domain and thus cannot initiate downstream signaling cascades, leads to targeting of IGF-2 to lysosomal degradation. Thus, IGF-2R functions to clear IGF-2 from the cell surface to attenuate signaling^17^,^18^. Here we further investigated the role of IGF-2 and its receptors on motor neurons and muscle to better understand oculomotor neuron resistance in ALS. Subsequently, we functionally tested the therapeutic benefit of IGF-2 delivery *in vitro* on human motor neurons derived from ALS patient fibroblasts. As we wanted to address if IGF-2 could be protective across diseases, we also treated human motor neurons derived from fibroblasts of spinal muscular atrophy (SMA) patients. SMA is a recessively inherited motor neuron disease that is caused by the loss of function of the Survival of Motor Neuron 1 (SMN1) protein^19^. While the underlying causes of ALS and SMA appear quite distinct, it has been shown that SMN, FUS and TDP-43 can functionally interact, indicating that SMA and ALS share pathways and supporting the view that common mechanisms could be targeted in these genetically distinct diseases^20^,^21^. This is further supported by the loss of spliceosome integrity that has been identified as a critical mechanism common to neurodegeneration in ALS and SMA^22^. Furthermore, mutant SOD1 has been shown to disrupt the recruitment of SMN1 to nuclear gems^23^. Finally, we delivered IGF-2 to SOD1^G93A^ fALS mice *in vivo* using adeno-associated virus 9 (AAV-9) to study effects on motor performance, life-span and motor neuron survival.

## Results

### IGF-2 was persistently expressed in oculomotor neurons in health and ALS

**Figure 1:**
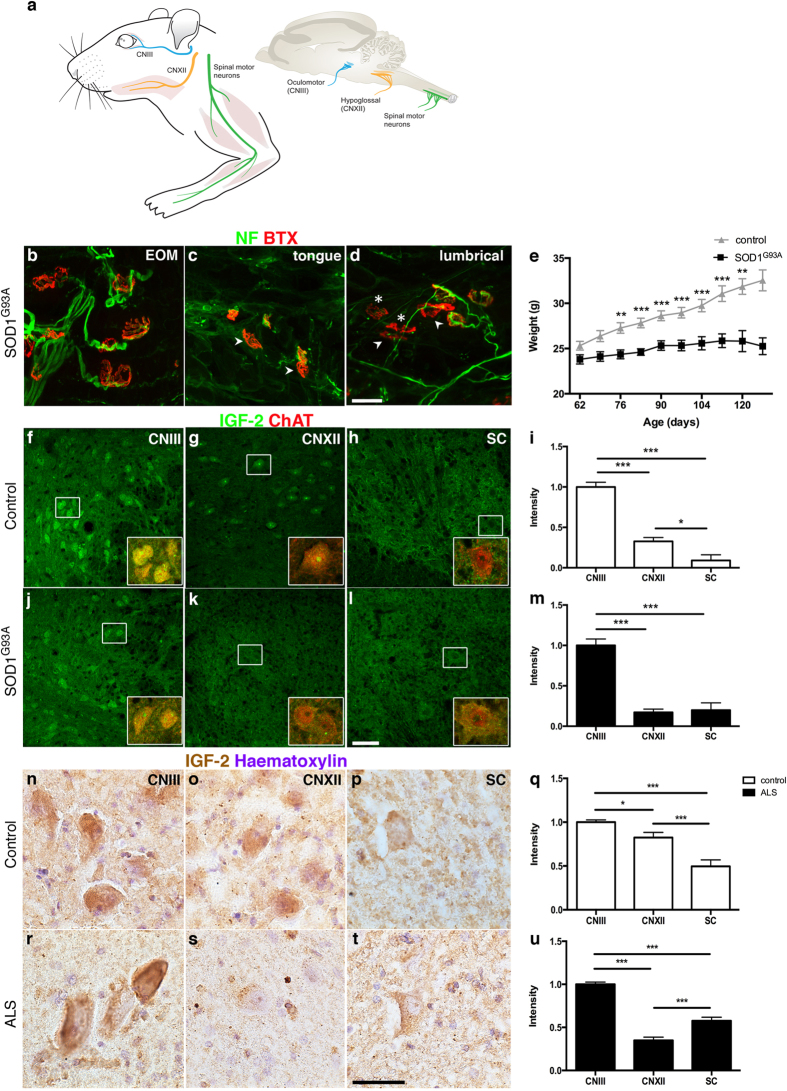
IGF-2 was persistently expressed in oculomotor neurons in health and ALS, in mouse and man. (**a**) Schematic of the central nervous system of the mouse and connected muscles, highlighting the location of the oculomotor neurons (CNIII, in blue) in the midbrain and their targets the extraocular muscles, which are relatively resistant to degeneration in ALS. Also depicted are the vulnerable hypoglossal motor neurons (CNXII, in yellow) and the tongue muscles they innervate and vulnerable spinal motor neurons in the ventral horn (in green), which innervate limb muscles (by Mattias Karlen). Neurofilament and SV2a stainings were used to visualize the presynaptic motor nerve and α-bungarotoxin (BTX) to label acetylcholine receptors (AChRs) on the muscle, showing that extraocular muscles (**b**) were fully innervated in symptomatic P126 SOD1^G93A^ mice, while tongue (**c**) and lumbrical muscles (**d**) showed partial (arrow head) or complete (*) denervation. (**e**) Weight curve of the fALS SOD1^G93A^ mouse model, showing the decrease in weight compared to wild-type littermates, characteristic for disease (*P* < 0.01 to *P* < 0.001, t(17) = 3.88–5.19), n = 8 control mice, n = 10 SOD1^G93A^ mice multiple t test). In P126 control mice, insulin-like growth factor-2 (IGF-2) protein was preferential to oculomotor neurons (**f**,**i**), with 2.7-fold higher levels than in hypoglossal (**g**,**i**) and 20-fold higher than spinal (**h**,**i**) motor neurons (F(2, 185) = 61.69, *P* < 0.0001, n = 4, ANOVA). IGF-2 levels were 7.6-fold higher in hypoglossal than in spinal motor neurons (*P* < 0.05, ANOVA). Analysis of P126 symptomatic SOD1^G93A^ mice showed that IGF-2 levels remained preferential to oculomotor neurons (**j**,**m**) with levels 4.1-fold higher than in hypoglossal (**k**,**m**) and 3.3-fold higher than in spinal motor neurons (**l**,**m**) (F(2, 241) = 36.05, *P* < 0.0001, n = 3, ANOVA). Analysis of non-demented control patient tissues showed that IGF-2 protein levels were higher in oculomotor motor neurons (**n**,**q**) compared to hypoglossal motor neurons ((**o**,**q**) F(2, 346) = 22.67, *P* < 0.05, n = 5, ANOVA; *P* < 0.0001, Kruskal-Wallis) and spinal motor neurons ((**p**,**q**) *P* < 0.0001, ANOVA; *P* < 0.0001, Kruskal-Wallis). IGF-2 remained preferential to oculomotor motor neurons in end-stage ALS patient tissue ((**r**–**u**) F(2, 500) = 98.78, *P* < 0.0001, n = 5, ANOVA; *P* < 0.0001, Kruskal-Wallis). Values in the graphs represent means ± SEM. Scale bar in (**d**) 30 μm (applicable to (**b**,**c**)), (**l**) 50 μm (applicable to (**f**–**h**,**j**,**k**), (**t**) 30 μm (applicable to (**n**–**s**)).

Oculomotor (CNIII) motor neurons in the midbrain and their targets, the extraocular muscles, are relatively resistant to degeneration in ALS while spinal motor neurons in the ventral horn of the spinal cord, which innervate limb muscles, are vulnerable (Fig. 1a)^7^,^8^,^9^,^11^,^24^,^25^. Analysis of muscle innervation in symptomatic P126 SOD1^G93A^ mice (a model of fALS^26^ based on over-expression of human mutated SOD1) found that extraocular muscles were still fully innervated at this time point (Fig. 1b), while tongue muscles, innervated by hypoglossal (CNXII) motor neurons, (Fig. 1c) and lumbrical muscles, innervated by lumbar spinal motor neurons, (Fig. 1d), showed evidence of denervation. The SOD1^G93A^ mice showed a decrease in weight from postnatal day 75 (P75) and onward, compared to wild-type littermate controls, characteristic for this model (Fig. 1e). To better understand the relative resistance of oculomotor neurons to degeneration in ALS, we now investigated the IGF-2 protein level in resistant and vulnerable motor neuron groups in rodent and human patient tissues, by quantifying signal intensity of IGF-2 immunostainings. Analysis of P126 control mice (from the SOD1^G93A^ colony) showed that the IGF-2 protein was higher in oculomotor neurons than in hypoglossal and spinal motor neurons (Fig. 1f–i), and remained preferential to oculomotor neurons in P126 symptomatic SOD1^G93A^ mice (Fig. 1j–m), at levels comparable to that seen in control mice (Supplementary Fig. 1a). Human post mortem analysis revealed that IGF-2 protein was also preferential to oculomotor neurons in non-demented control tissues compared to hypoglossal and spinal motor neurons (Fig. 1n–q). Importantly, IGF-2 remained preferential to oculomotor neurons in end-stage ALS patient tissue (Fig. 1r–u), indicating that this growth factor could play a protective role in these resistant motor neurons in disease.

### IGF-1R and IGF-2R expression was predominant in resistant oculomotor neurons and extraocular muscles

**Figure 2:**
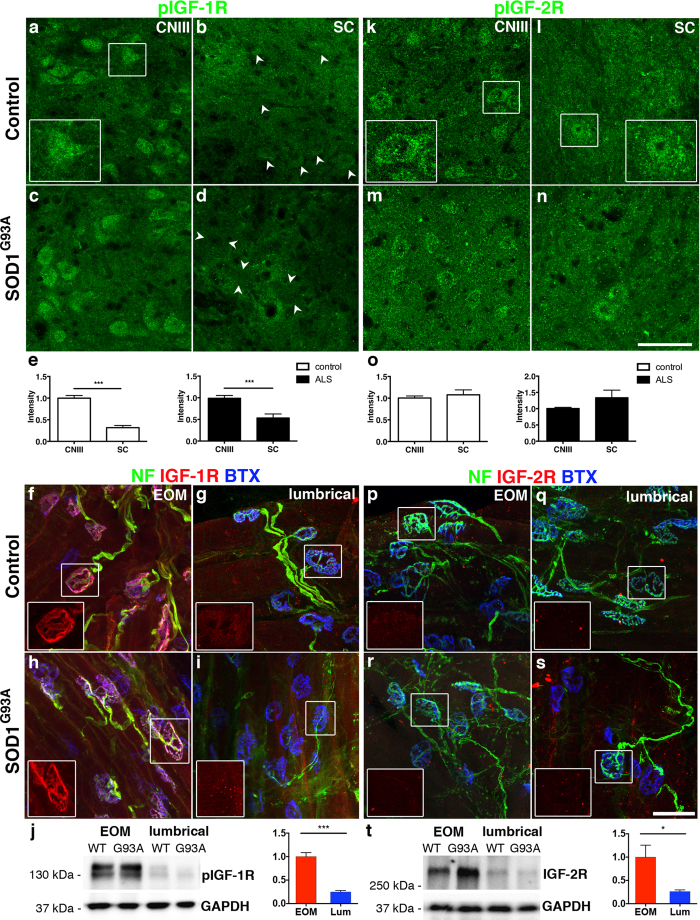
IGF-1R and IGF-2R expression was predominant in resistant oculomotor neurons and extraocular muscles. Phosphorylated IGF-1R (pIGF-1R) protein was present in oculomotor motor neurons in wild-type and SOD1^G93A^ mice (**a**,**c**,**e**), with signficantly lower levels in motor neurons in spinal cord in control (**b)** arrow heads, (**e**) t(113) = 8.69, n = 4 mice, *P* < 0.0001, Student’s t test, values represent means ± SEM) and SOD1^G93A^ mice ((**d**) arrow heads, (**e**) t(141) = 4.30, *P* < 0.0001, n = 5 mice, Student’s t test, values represent means ± SEM). IGF-1R protein was strongly expressed and co-localized with endplates in extraocular muscles in wild-type and SOD1^G93A^ mice (**f**,**h**), while it was barely detectable in lumbrical muscles (**g**,**i**). Western blot analysis confirmed that the pIGF-1R protein level was 4-fold higher in extraocular muscles than in lumbrical muscles ((**j**) t(8) = 8.20, n = 5 mice, *P* < 0.0001, Student’s t test, values represent means ± SEM). Phosphorylated IGF-2R (pIGF-2R) protein was present at comparable levels in oculomotor (**k**,**m**) and spinal (**l**,**n**) motor neurons in both wild-type ((**o**) t(92) = 0.6829, *P* = 0.4964, n = 3 mice, Student’s t test) and SOD1^G93A^ mice ((**o**) t(102) = 1.880, *P* = 0.0630, n = 3 mice, Student’s t test). Peripherally, IGF-2R protein was barely detectable in extraocular muscles (**p**,**r**) and undetectable in lumbrical muscles (**q**,**s**) of wild-type and SOD1^G93A^ mice using immunofluorescence. Western blot analysis showed that IGF-2R was indeed present in extraocular muscles at 3.8-fold higher levels than in lumbrical muscles ((**t**) t(8) = 2.86, *P* = 0.021, n = 5 mice/group, Student’s t test, values represent means ± SEM). Scale bars: (**p**) 50 μm (applicable to (**a**–**d**) and (**m**–**o**)), (**q**) 40 μm (applicable to (**e**–**h**) and (**n**–**q**)).

IGF-2 exerts its biological actions through binding to IGF-1R (survival or proliferation) or insulin receptors (proliferation), while binding to IGF-2R leads to endosomal degradation^17^. In this study we focused on IGF-1R and IGF-2R expression centrally on motor neurons and peripherally in neuromuscular junctions (NMJs). Immunofluorescent analysis using an antibody against phosphorylated IGF-1R (pIGF-1R) protein showed a high level of activated IGF-1R within oculomotor neurons in wild-type and symptomatic SOD1^G93A^ mice (Fig. 2a,c,e), with much lower levels on spinal motor neurons (Fig. 2b,d,e). The level of pIGF-1R was comparable between control and SOD1^G93A^ mice (Supplementary Fig. 1b). Using an antibody against both phosphorylated (active) and non-phosphorylated (inactive) IGF-1R gave a similar result to the pIGF-1R antibody staining (Supplementary Fig. 1c–g). Furthermore, in SOD1^G93A^ spinal cords, most pIGF-1R staining was present in close proximity to motor neurons and appeared associated with glial cells (Supplementary Fig. 1h,i). IGF-1R protein also co-localized with NMJs in extraocular muscles in wild-type (Fig. 2f) and SOD1^G93A^ mice (Fig. 2h), while it appeared almost absent from lumbrical muscles (Fig. 2g,i). Western blot analysis using two antibodies specific for distinct phosphorylation sites on IGF-1R (Supplementary Fig. 1j) confirmed the data from the immunofluorescent analysis and showed that extraocular muscles contained 4-fold higher levels of activated IGF-1R than lumbrical muscles (Fig. 2j, *P* < 0.0001). High magnification confocal images of IGF-1R staining in neuromuscular junctions in extraocular muscles (Supplementary Fig. 2a–i) clearly indicated that IGF-1Rs were present on both the pre-terminal axon (Supplementary Fig. 2g) and more prominently postsynaptically on the muscle endplate (Supplementary Fig. 2h,i). Phosphorylated IGF-2R (pIGF-2R) protein was present at comparable levels in motor neurons of the oculomotor nucleus (Fig. 2k,m) and spinal cord (Fig. 2l,n) in wild-type and SOD1^G93A^ mice. However, the level of pIGF-2R within oculomotor neurons was slightly decreased in the SOD1^G93A^ mice compared to control (Supplementary Fig. 1k). Peripherally, IGF-2R protein was barely detectable in extraocular (Fig. 2p,r) or lumbrical muscles (Fig. 2r,s) using immunofluorescence. However, western blot analysis showed that IGF-2R protein was indeed present in extraocular muscles, at significantly higher levels than in lumbrical muscles (Fig. 2t, *P* = 0.021). In summary, the presence of high levels of phosphorylated IGF-1R protein on oculomotor neurons and extraocular NMJs indicate that IGF-2 could exert a positive effect both centrally and peripherally on these resistant motor neurons.

### IGF-2 protected human spinal motor neurons from ALS-like toxicity *in vitro*

**Figure 3:**
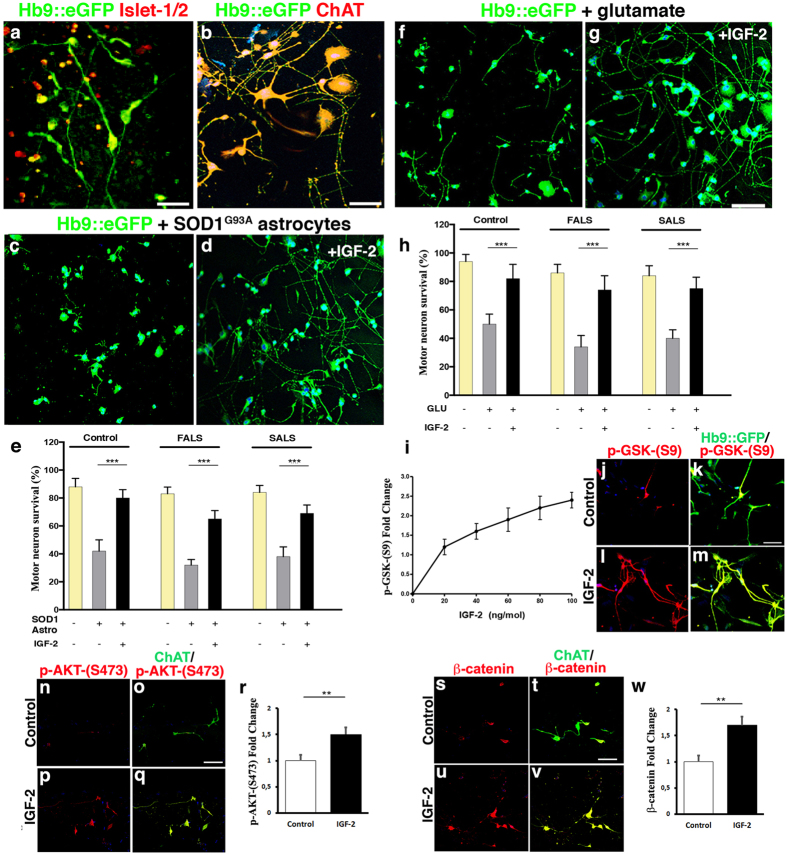
IGF-2 protected human spinal motor neurons from ALS-like toxicity *in vitro*. Human spinal motor neurons generated from iPSCs expressed Islet-1/2 (**a**) and ChAT (**b**) (photos of iPSC clone AM/ALS1.1 shown, see supplementary Table 1). The number of motor neurons was significantly decreased after co-culture with SOD1^G93A^ astrocytes ((**c**–**e**), F(8, 126) = 231.02, *P* < 0.0001, ANOVA). Addition of IGF-2 (50 ng/ml) 24–48 after initiation of toxicity could rescue motor neurons ((**d**,**e**), F(8, 126) = 231.02, *P* < 0.0001, ANOVA). Exposure of cultures to glutamate also induced motor neuron degeneration ((**f**,**h**), F(8, 126) = 125.87, *P* < 0.001, ANOVA), which could be rescued by adding IGF-2 (treatment initiated 24–48h after addition of glutamate) ((**g**,**h**), F(8, 126) = 125.87, *P* < 0.0001, ANOVA). Values represent means ± SD from 5 independent experiments performed in triplicate. Addition of IGF-2 induced phosphorylation of GSK-3 on the S9 residue p-GSK-(S9), thus inhibiting enzyme activity in a dose-dependent way (**i**). Representative images showing p-GSK-(S9) levels in the absence (**j**,**k**) or presence (**l**,**m**) of IGF-2. Representative images depicting AKT activation by phosphorylation on residue S473 (p-AKT-(S473)), in the absence (**n**,**o**) or presence (**p**,**q**) of IGF-2. IGF-2 induced p-AKT-(S473) levels in iPSC motor neurons ((**r**), t(8) = 5.99, *P* < 0.001, n = 5, Student’s t test, values represent means ± SD). Immunofluorescent analysis of β-catenin levels in control (**s**,**t**) and IGF-2 treated (**u**,**v**) motor neurons. IGF-2 induced β-catenin levels in iPSC motor neurons ((**z**), t(8) = 6.36, *P* < 0.001, n = 5, Student’s t test, values represent means ± SD). Scale bars: (**a**) 60 μm, (**b**) 50 μm, (**g**) 75 μm (applicable to (**c**,**d**,**f**)), (**k**) 75 μm (applicable to (**j**,**l**,**m**)), (**o**) 50 μM (applicable to (**n**,**p**,**q**), (**t**) 50 μM (applicable to (**s**,**u**,**v**)).

We now asked if IGF-2 could prevent ALS-like degeneration of human motor neurons derived from induced pluripotent stem cells (iPSCs). Even if motor neurons derived from sALS and fALS patient iPSCs display some key hallmarks of the human disease *in vitro*, such as TDP-43 aggregates and C9ORF72 dipeptides, motor neuron cell degeneration in culture is not overt, in particular in sALS MNs^27^. Therefore, to model ALS *in vitro*, we used two different assays; glutamate excitotoxicity^11^,^28^ or co-culture with SOD1^G93A^ astrocytes, which are selectively toxic to motor neurons^29^,^30^,^31^ (Supplementary Fig. 3a). Spinal motor neurons were differentiated from the iPSCs and monitored using a motor neuron specific lenti-Hb9::*eGFP* construct. The susceptibility of iPSCs-derived motor neurons to glutamate overload, which could be a general downstream event in motor neuron disease, was determined over a range of concentrations (1 to 100 μM). For the ALS astrocyte toxicity test, iPSCs-derived motor neurons were plated on the bottom chamber of a transwell co-culture system and cultured with astrocytes (in the upper compartment) obtained from SOD1^G93A^ mice. The porous membrane that separates the two compartments only allows diffusion of soluble molecules, allowing testing of non autonomous cell death. Control motor neurons degenerated in response to co-culture with SOD1^G93A^ astrocytes (Supplementary Fig. 3b) and showed susceptibility to glutamate (Supplementary Fig. 2c). Addition of IGF-2 alleviated glutamate toxicity in a dose-dependent manner when administered prior to induction of toxicity (Supplementary Fig. 3d). sALS and fALS patient motor neurons were more sensitive to glutamate and astrocyte toxicity than control motor neurons (Supplementary Fig. 3e,f). Pretreatment with IGF-2 protected motor neurons in both ALS-toxicity assays (Supplementary Fig. 3g,h). Prior to toxicity, motor neurons (expressing a lenti-Hb9::*eGFP* construct) appeared healthy, extended processes and expressed Islet-1/2 (Fig. 3a) and ChAT (Fig. 3b). Importantly, addition of IGF-2 one to two days after initiation of toxicity also protected motor neurons from SOD1^G93A^ astrocytes (Fig. 3c–e) and glutamate over-load (Fig. 3f–h). To identify molecular targets of IGF-2 in the protection of ALS motor neurons we examined the effect of IGF-2 on site-specific phosphorylation of glycogen synthase kinase 3 beta (GSK3β). IGF-2 is an activator of the PI3K/Akt pathway, through IGF-1R binding, which can regulate neuronal survival through phosphorylation and thereby inhibition of GSK3β^32^,^33^. Levels of GSK3β Ser9 phosphorylation (p-GSK-(S9)) were strongly elevated in response to IGF-2 in a dose-dependent manner (Fig. 3i–m), indicating that GSK3β was inhibited. We could also demonstrate that IGF-2 treatment increased the level of Akt phosphorylated on residue Ser473 (p-AKT -(S473)) in iPSC motor neurons (Fig. 3n–r), and thus a higher degree of PI3/Akt pathway activation. Finally, we showed that IGF-2 induced an upregulation of β-catenin levels in iPSC motor neurons (Fig. 3s–w), thus confirming the activation of the PI3/Akt and inhibition of GSK3β. Collectively, our data demonstrate that IGF-2 can protect ALS patient motor neurons from degeneration in two different ALS-like toxicity systems and that PI3/Akt activation and subsequent GSK3β inhibition, mediated through IGF-1R binding, may in part mediate this protective effect.

### IGF-2 protected SMA patient spinal motor neurons in culture

**Figure 4:**
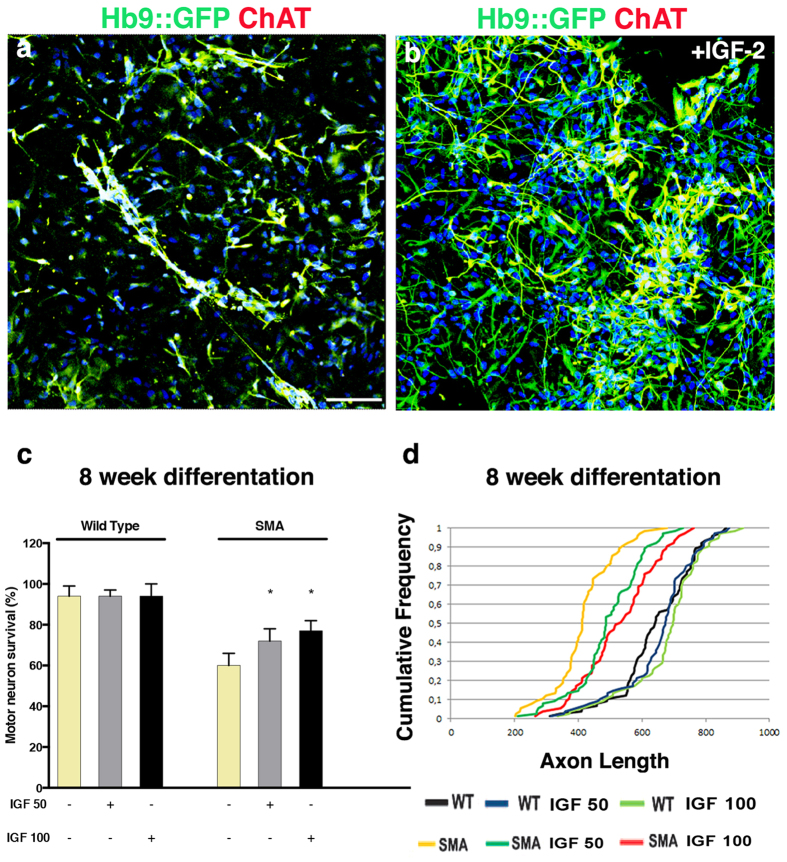
IGF-2 protected SMA patient spinal motor neurons in culture. SMA patient motor neurons in long-term culture without (**a**) or with (**b**) IGF-2 (photos of iPSC clone SMA 1.1 shown, see supplementary Table 2). (**c**) The number of SMA motor neurons in culture was significantly decreased compared to wild-type cells (F(5, 84) = 135,65, *P* < 0.0001, ANOVA). Treatment of the cultures with IGF-2 (50–100 ng/ml, added at 4 weeks) was protective to motor neurons (8 weeks, grey and black bars, F(5, 84) = 135.65, *P* < 0.0001, ANOVA). Values represent means ± SD from 5 independent experiments performed in triplicate. (**d**) At 8 weeks, untreated SMA iPSC motor neurons (shown in yellow) showed shorter axon lengths than wild-type cells (shown in black). SMA motor neurons treated with IGF-2 (shown in teal and red) had longer axons than untreated SMA motor neurons (*P* < 0.001, Kolmogorov-Smirnov test, 5 independent experiments performed in triplicate).

To test if IGF-2 could also protect against degeneration across motor neuron diseases, we used motor neurons derived from SMA patient iPSCs, which degenerate due to a lack of SMN1 protein. SMA iPSCs generate motor neurons at a normal rate, but these motor neurons present an apparent cell autonomous degeneration *in vitro*, which is evident after 8 weeks of culturing^5^,^34^ (Fig. 4a,c). When IGF-2 was added to the culture at 4 weeks and subsequently maintained, motor neurons were protected and showed significantly improved survival at 8 weeks of culture (Fig. 4a–c). Furthermore, morphometric analysis showed that IGF-2-treated SMA patient motor neurons had increased neurite lengths compared to untreated motor neurons (Fig. 4d). In conclusion, our data shows that IGF-2 can also protect human motor neurons from SMA-induced degeneration *in vitro*. The regenerative properties exerted by IGF-2 on human motor neurons indicate that it could elicit beneficial effects on nerve growth of motor neurons that have lost connection with muscle.

### IGF-2 prolonged the survival of SOD1^G93A^ fALS mice by preserving motor neurons and inducing nerve regeneration

**Figure 5:**
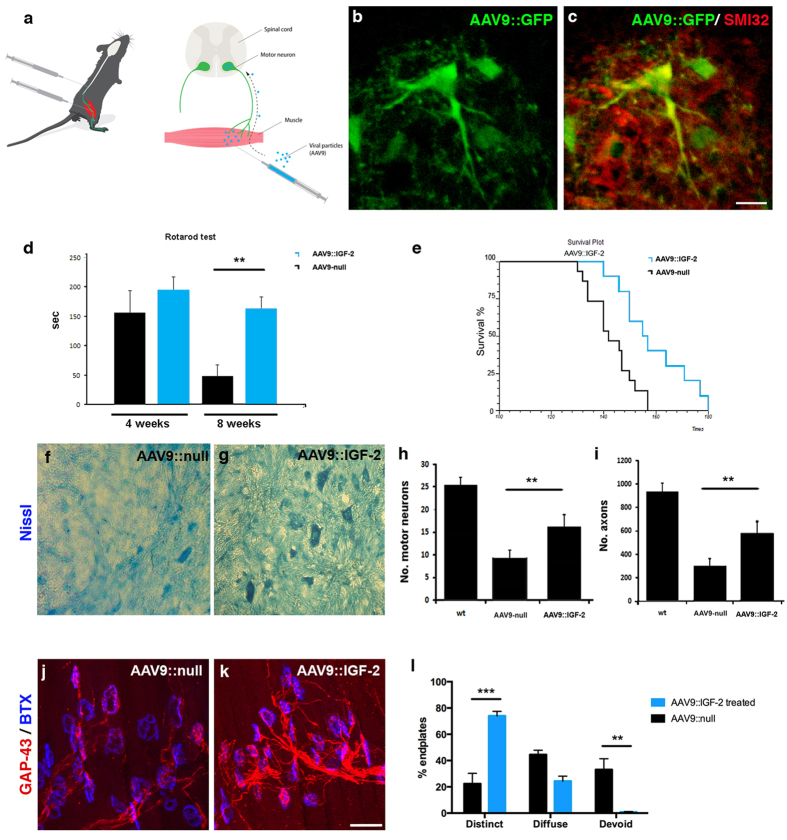
IGF-2 prolonged the survival of SOD1^G93A^ fALS mice by preserving motor neurons and inducing nerve regeneration. (**a**) Schematic drawing of the IGF-2 *in vivo* delivery (by Mattias Karlen) where SOD1^G93A^ mice were injected into the hindlimb quadriceps and thoracic muscles at 80 days of age with AAV9::*GFP*, AAV9::*IGF*-*2* or AAV9::*null* at a total dose of 11 × 10^11^ vg. (**b**) Injection of AAV9::*GFP* resulted in GFP expression within the spinal cord 2 weeks post-injection. (**c**) Colocalization of GFP (green) with SMI32 (red) demonstrated that motor neurons were efficiently transduced (n = 5 mice). (**d**) Rotarod performance of AAV9::*IGF*-*2* treated mice (n = 10) was significantly improved compared to AAV9::*null* mice (n = 10) in particular at 8 weeks after treatment (F(1, 36) = 18.66, *P* < 0.0001, two-way ANOVA; at 8 weeks: T(16) = 4.778, *P* < 0.001, Student’s t test). Error bars indicate mean ± SEM). (**e**) Kaplan-Meier survival curves demonstrated a significantly extended survival by 14 days in AAV9::*IGF*-*2* mice (n = 10) compared to AAV9::*null* animals (*n* = 15) (*χ*^2^  =  5.3, *P* = 0.02). Representative motor neuron pools in the lumbar segment of the spinal cords of AAV9::*null* (**f**) and AAV9::*IGF*-*2* (**g**) treated mice at P140. Quantification of motor neurons (**h**) and axons (**i**) in the lumbar spinal cords of AAV9::*IGF*-*2* and AAV9-null treated mice (mean ± SD) at P140. Motor neuron and axon counts significantly increased in the AAV9::*IGF*-*2* treatment group compared to the AAV9::*null* group (motor neurons: F(2, 87) = 397.75, *P* < 0.0001; axons: F(2, 33) = 230.28, *P* < 0.0001, two-way ANOVA, n = 3/group). (**j–l**) Analysis of GAP-43 in NMJs of lumbrical muscles showed that AAV9::*IGF*-*2* treatment significantly increased the proportion of endplate with distinct GAP-43 expression in SOD1^G93A^ mice compared to AAV9::*null* treatment *F*(2, 25) = 38.07, *P* < 0.0001, n = 5 muscles (3 mice) AAV9::*null*, n = 6 muscles (3 mice) AAV9::*IGF*-*2*, ANOVA, values shown as mean ± SEM). (**j**–**l**) AAV::*IGF*-*2* treated mice also had significantly fewer endplates which were devoid of GAP-43 staining (*P* < 0.001, ANOVA, values shown as mean ± SEM). Scale bar: (**c**) 50 μm (applicable to (**b**)), (**k**) 40 μm (applicable to (**j**)).

Based on the positive effects of IGF-2 on motor neuron somas and axons, we next investigated if administration of IGF-2 could extend the life-span of SOD1^G93A^ fALS mice. Here, an AAV9 vector expressing *IGF*-*2* was injected bilaterally into the hindlimb quadriceps and thoracic muscles at P80 (11 × 10^11^ particles in total). An AAV9::*null* vector was used as a control for survival experiments and an AAV9::*GFP* vector to show transduction efficiency of motor neurons (Fig. 5a). Using the AAV9::*GFP* vector we could demonstrate that the virus transduced the spinal cord, including motor neurons, extensively (Fig. 5b,c). Importantly, AAV9::*IGF*-*2* treated SOD1^G93A^ mice showed significant improvement in their neuromuscular function, in particular at 8 weeks post injection, as assessed by rotarod performance (Fig. 5d). Furthermore, AAV*9*::*IGF*-*2* treated SOD1^G93A^ mice showed extended survival by 14 days (157 ± 10 days mean survival) compared to AAV9::*null* treated SOD1^G93A^ mice (143 ± 5 days mean survival) (Fig. 5e, *χ*^2^ = 5.3, *P* = 0.02). Histological evaluation of the lumbar spinal cord revealed that AAV9::*IGF*-*2* treatment prevented the pathological changes leading to a qualitative reduction in neuropil and cellular vacuolization in animals at 140 days of age (Fig. 5f,g). To determine if the positive effects exerted by AAV9::*IGF*-*2* was due to a preservation of motor neurons, we quantified the number of motor neuron somas in spinal cord sections and axons in ventral spinal nerve roots. At P140, motor neuron loss was significantly decreased by AAV9::*IGF*-*2* administration (Fig. 5h). IGF-2 treated SOD1^G93A^ mice also showed preservation of axonal density in the L4 ventral root compared to AAV9::*null* injected mice (Fig. 5i). IGF-2 can induce motor nerve sprouting^35^ and some motor neurons show a great capacity for sprouting and regeneration in the SOD1^G93A^ mouse^36^,^37^,^38^,^39^,^40^. Therefore we next investigated if our IGF-2 treatment had induced regeneration in the lumbrical muscles in the hind paws of treated mice. NMJs were quantified based on their expression of GAP-43, a marker of regenerating neurons (Supplementary Fig. 4a–c). Importantly, NMJs in AAV9::*IGF*-*2* treated muscles contained a significantly larger proportion of endplates with distinct GAP-43 expression compared to AAV9::*null* treated mice (Fig. 5j–l) and had significantly fewer endplates which were devoid of GAP-43 staining (Fig. 5j–l). In conclusion, our data demonstrates that AAV9::*IGF*-*2* delivery induced a regenerative response in spinal motor neurons in these mice, which likely in part accounts for the observed functional improvement.

## Discussion

In this report we show that the neurotrophic factor IGF-2 was predominantly and persistently expressed in oculomotor motor neuron cell bodies in symptomatic SOD1^G93A^ mice and end-stage ALS patients, indicating that IGF-2 could play a part in protecting these cells. Furthermore, the high level of IGF-1R protein on oculomotor neurons and postsynaptically on extraocular muscle endplates indicate that IGF-2 could exert a positive effect both centrally and peripherally on these motor neurons. Addition of IGF-2 to the culture media protected both ALS and SMA patient motor neurons from degeneration. Finally, viral-mediated delivery of IGF-2 to SOD1^G93A^ ALS mice improved motor performance, increased the life-span and protected motor neurons cell bodies, axons and promoted motor nerve regeneration.

Identification of transcript and proteins specifically enriched or lacking in oculomotor neurons^11^,^12^,^13^,^41^,^42^ could give clues to their intrinsic resistance to degeneration in ALS, and perhaps across motor neuron diseases. Here, we show that IGF-2 and phosphorylated IGF-1R were present on oculomotor neuron somas. This indicates that IGF-2 and/or IGF-1 has bound to and activated the receptor on the cell surface. From our studies we can not discern if IGF-1R receptor activation on oculomotor neurons was induced through cell autonomous secretion of IGF-2, or through IGFs produced by the choroid plexus^43^ and circulating in the cerebrospinal fluid (CSF). However, the specific IGF-1R activation on oculomotor neurons and much lower level of pIGF-1R on spinal motor neurons, indicate that local mechanisms are in play, presumably through IGF-2 production from oculomotor neurons themselves. Nonetheless, the presence of high levels of pIGF-2R on both spinal and oculomotor neurons indicate that endosomal degradation pathways are needed more in general and not just on resistant motor neurons, presumably through circulating IGFs. The strong expression of pIGF-2R in spinal motor neurons is consistent with a previous study in rat which showed prominent expression in spinal and facial motor neurons^44^, while oculomotor expression was not investigated.

We also identified IGF-1Rs in NMJs of extraocular muscles. Here the staining was partly overlapping with the neurofilament marker SV2a, indicating that IGF-1R was present presynaptically on motor neurons. However, the most prominent expression was present postsynaptically on the muscle endplates as evident by co-localization with BTX. The low expression of IGF-2 receptors on muscle at this age is a clear reflection of the low endogenous production of IGF in adult muscle. Combined with the highly localized IGF-1R expression on motor endplates, this indicates that IGFs released from presynaptic terminals of oculomotor neurons act on receptors on muscle and that this interaction could exert a positive effect on motor neuron-muscle connectivity. Indeed, we found that the extraocular NMJs were stable in late symptomatic ALS mice, in contrast to vulnerable NMJs which were remodeled as visualized by GAP-43 staining.

Encouragingly, our *in vitro* experiments showed that IGF-2 was protective to motor neurons derived from fALS, sALS and SMA patients. Here we used established assays of motor neuron disease, utilizing either glutamate overload or mutant SOD1 astrocyte-induced toxicity to model ALS^11^,^27^,^29^,^30^ and the accelerated cell intrinsically-mediated cell death of SMA patient motor neurons^5^,^34^. The protective effect of IGF-2 appeared independent of the cause of degeneration, which is promising for future therapeutic purposes, as the pathways of motor neuron degeneration in ALS could vary from case to case. Importantly, IGF-2 was also protective when added after induction of toxicity, which is clinically more relevant than prior treatment. IGF-2 has been shown to be beneficial for axon sprouting in the mouse^35^,^45^. In addition to improving survival, IGF-2 treatment increased neurite lengths of SMA iPSC motor neurons. Furthermore, we showed that IGF-2 treatment activated PI3K/Akt signaling in motor neurons as evidenced by increased levels of phosphorylated Akt and increased GSK3β phosphorylation, which inhibits GSK3 activity. IGF-2 treatment also affected downstream signaling of GSK3β as demonstrated by the upregulation of β-catenin expression. Importantly, β-catenin has a critical role in axonal and dendrite extension and maintenance^46^. This indicates that the protective effect of IGF-2 was mediated by GSK inhibition through the PI3K-Akt pathway. This is in agreement with a previous study which identified kenpaullon, a GSK inhibitor, as a survival factor for ALS patient iPSC motor neurons^47^.

During development, IGF-1 acts as a target-derived trophic factor for oculomotor neurons, promoting both survival and axon outgrowth^48^, presumably by acting on IGF-1R on oculomotor neurons, as does IGF-2. In our culture system, the addition of IGF-2 to the media could be seen as mimicking such muscle-secretion of a motor neuron survival factor rather than demonstrating a cell intrinsic survival factor. Nonetheless, this implies that it could be sufficient to deliver IGF-2 to muscle, without retrograde delivery, to protect motor neurons that are still connected with muscle or within close enough range to benefit from secreted IGF-2, especially as motor neurons show regenerative capacity also in ALS patients^40^. However, it is likely more beneficial to deliver IGF-2 also to motor neurons, through retrograde delivery, as in our *in vivo* approach, or through direct injection into spinal cord to target motor neurons that have retracted further from the muscle. This notion is supported by the previous finding that muscle-restricted expression of IGF-1 was less protective in SOD1^G93A^ mice compared AAV-based retrograde delivery of IGF-1, which reached motor neuron somas^16^. Even though spinal motor neurons had a much lower level of IGF-1R compared to oculomotor neurons, this was apparently sufficient to induce a protective response after exogenous administration of IGF, as shown by the successful use of IGF-1 in SOD1^G93A^ mice^16^ and using IGF-2 in our study. Specifically, delivery of AAV2::*IGF*-*1* to 60-day old presymptomatic SOD1^G93A^ mice delayed the median onset by 31 days, with a survival that was 37 days longer than GFP-treated mice. When the virus was instead delivered to 90-day-old SOD1^G93A^ mice the median survival was extended by 22 days^16^. In our study, IGF-2 delivery to 80-day-old SOD1^G93A^ mice, which display extensive muscle denervation^6^, prolonged the life-span by 14 days. The somewhat smaller effect seen with our IGF-2 treatment could be due to the lower affinity of IGF-2 for the IGF-1R compared IGF-1. It could also be the result of a less efficient retrograde transport of AAV9 than AAV2 to motor neurons somas, but this remains to be further investigated. IGF-1 delivery to motor neurons^49^ or muscle^50^ in SMA mice can also be protective. However, the broadly increased IGF-1R levels seen in SMA mouse spinal cords and the protective effects demonstrated by a general genetic reduction of IGF-1R^51^, warrants for cell-specific over-expression of IGFs in this model.

Several clinical trials of subcutaneously delivered IGF-1 in ALS have been conducted with contradictory results^52^,^53^,^54^. IGF-2 has not yet been tested in clinical trials for ALS. We anticipate that delivery of IGF-2 or IGF-1 directly to motor neurons, using gene therapy, either through muscle injections and retrograde delivery to motor neuron somas or intraspinal injections, could confer localized neurotrophic support directly to motor neurons and be beneficial to ALS patient.

Our gene therapy data showed that IGF-2 prolonged the life-span of the SOD1^G93A^ mice, preserving both motor neuron somas and axons. We showed that IGF-2 induced a significant regenerative response *in vivo*, demonstrating the capability of motor neurons to regenerate in symptomatic animals. In ALS, some motor axons show great capacity for regeneration and appear to compensate for the loss of innervation by their neighbors^36^,^38^. Thus, factors that influence motor nerve regeneration could determine motor neuron and muscle connectivity and consequently disease onset and duration. This is clearly illustrated by the ephrin receptor EphA4, which mediates axon repulsion and is a disease modifier in ALS^55^. It is possible that combining IGF-2 treatment with blocking of EphA4 could further improve the axon regrowth and connectivity with muscle in ALS.

In summary, our findings support a general motor neuron protective role for IGF-2 in ALS and indicate that the higher level of IGF-2 and IGF-1Rs in oculomotor motor neurons and endplate on extraocular muscles could be protecting these motor neurons against degeneration. Finally, our results demonstrate that oculomotor-specific expression can be utilized to identify candidates that protect vulnerable motor neurons from degeneration in ALS and that such candidates can have protective potential across motor neuron diseases.

## Methods

### Ethics statement

All the work involving animal or human subjects/tissues was carried out in accordance with the Code of Ethics of the World Medical Association (Declaration of Helsinki) and with national legislation and institutional guidelines. Animal procedures were approved by the Swedish animal ethics review board (Stockholms Norra Djurförsöksetiska nämnd) and Italian Ministry of Health review boards. Ethical approval for the use of the human post mortem samples was obtained from the regional ethical review board in Stockholm, Sweden (Regionala Etikprövningsnämnden, Stockholm, EPN). All post mortem human tissues were obtained from the Netherlands Brain Bank (NBB, www.brainbank.nl) or the National Disease Research Interchange (NDRI, www.ndriresource.org) with the written informed consent from the donors or the next of kin. Human fibroblasts were retrieved from Eurobiobank with informed consent (ethical committee approved at the IRCCS Foundation Ca’ Granda Ospedale Maggiore Policlinico).

### Generation of AAV vectors

Adeno-associated virus (AAV) vectors were manufactured by SignaGen Laboratories (www.signagen.com). The human IGF-2 cDNA (NCBI accession number BC000531) was cloned into a shuttle plasmid containing both the AAV2 inverted terminal repeats (ITR) and the 1.6-kb cytomegalovirus (CMV) enhancer/chicken β-actin (CBA) promoter. AAV9::*IGF*-*2* was produced by transient transfection of HEK293 cells using a double-stranded AAV2-ITR-based CBA vector with a plasmid encoding Rep2Cap9 along with an adenoviral helper plasmid (pHelper; Stratagene). Our serotype 9 sequence was verified by sequencing. We used AAV9 encoding GFP under the control of the CMV promoter (AAV9::*GFP*) to monitor transduction efficacy. As a control, a third vector was generated in which the IGF-2 cDNA was replaced with a noncoding sequence under the CBA promoter to generate the AAV9::*null* vector.

### Animal models

Adult male and female SOD1^G93A^ mice (B6.Cg-Tg(SOD1-G93A)1Gur/J)^26^ were used as a model of ALS and non-transgenic littermates were used as controls. Time points used were P112, P126, P140 and end-stage (individually detailed within results). Animals were housed according to standard conditions, with access to food and water ad libitum and a dark/light cycle of 12 h.

### Processing and immunohistochemistry of mouse and human tissues

#### Mouse immunohistochemistry

SOD1^G93A^ mice and wild-type littermates were sacrificed by inhalation of CO_2_ for muscle analysis. The extraocular muscles and lumbrical muscles (from the plantar surface of the hind-paw) were dissected in 0.1 M PBS and fixed in 4% PFA (Sigma-Aldrich) for 10 min for NMJ analysis or snap frozen in 2-Methylbutane (Sigma-Aldrich) on dry ice for immunoblotting. Only muscles innervated by CNIII were included in the extraocular analysis (superior rectus, inferior rectus, medial rectus, and inferior oblique). Staining for NMJ analysis was done as previously described^6^ using antibodies detailed in Table 1. For CNS immunohistochemistry, animals were anesthetized with avertin (2,2,2-Tribromoethanol; Sigma-Aldrich) and perfused intracardially with PBS followed by 4% PFA. Brains and spinal cords were dissected and postfixed (for 3 hours and 1 hour, respectively), cryoprotected in sucrose and sectioned (30 μm). All CNS tissues were stained as previously described^12^ using the following primary antibodies (Table 1) and counter stained with NeuroTrace 435/455 Blue Fluorescent Nissl Stain (1:200 in PBS; Life Technologies) for 30 min. Tissues were imaged on a Zeiss LSM700 confocal microscope.

#### Immunohistochemistry of human tissues

The characteristics of ALS patients and non-demented controls (ND) used for immunohistochemical analysis are listed in Supplementary Table 1. Tissues were processed and subjected to immunohistochemistry as previously described^12^, using the following primary antibodies (Table 1). Brightfield images were captured using a Zeiss Axio Imager M1 Upright microscope.

#### Motor neuron intensity and area measurements

Signal intensities of IGF-2 and IGF receptor stainings were measured as described previously^12^. To aid human and mouse tissue analysis adjacent sections were stained with Nissl or antibodies against ChAT, allowing us to easily visualize motor neurons. Omission of either primary or secondary antibodies did not result in significant background levels (data not shown). All intensity measurements were normalized to the oculomotor nucleus. All quantifications were performed blind to the genetic status of the material.

### Western blot analysis of IGF-1 and IGF-2 receptor levels

Tissues were homogenized in ice-cold modified RIPA buffer (50 mM Tris HCl, 1% triton X-100, 0.5% Na deoxycholate, 0.2% SDS, 100 mM NaCl, 1 mM EDTA, pH7.5) with 0.4 mM PMSF and a protease inhibitor cocktail (complete, Mini, EDTA-free, Roche) using an electric tissue homogenizer (TissueRuptor, Qiagen) for 15 seconds followed by incubation on ice for 30 min. Samples were sonicated briefly (5 seconds) and centrifuged for 10 min at 4 °C and 13,200 rpm. Protein concentrations of the supernatants were determined with the Pierce BCA Protein Assay (Thermo Scientific). Samples were diluted with 4x Laemmli loading buffer with 10% mercaptoethanol and incubated on a shaker at 70 °C for 10 min before loading 20 μg of protein onto 3–8% Tris Acetate gradient gels (LifeTechnologies). Transfer was done in Bjerrum buffer with 10% Methanol onto a PVDF membrane for 1 hour at 30 V. After transfer, membranes were kept in 0.1% TBS-T or 0.5% PBS-T at 4 °C until further processing. Membranes were blocked in 1% BSA in 0.1% TBS-T or 5% BSA in 0.5% PBS-T for 1 hour followed by incubation with primary antibodies (Table 1). After washing, membranes were incubated with HRP-conjugated secondary antibodies (goat anti rabbit-HRP, 1:10,000; Dako) and proteins were visualized using enhanced chemiluminescence (Amersham ECL Prime Western Blotting Detection Reagent, GE Healthcare) followed by imaging in a Molecular Imager ChemiDoc XRS+ (BioRad).

#### Quantification of western blots

Band intensities were measured using ImageJ software. IGF-1R and IGF-2R expression is shown relative to GAPDH expression.

### iPSC lines and motor neuron cultures

#### Differentiation of human iPSCs into motor neurons

iPSC lines were generated from fibroblasts obtained from Eurobiobank, see Supplementary Table 2. The cells were tested for Mycoplasma (MycoAlert kit, Lonza). Spinal motor neurons were differentiated using a protocol developed for human embryonic stem cells and iPSCs^56^. For the generation of motor neurons from ALS, SMA and control patient iPSCs, cells were plated with neuronal medium composed of DMEM/F12 (Life Technologies), supplemented with MEM nonessential amino acids solution (Life Technologies), N2 (Invitrogen), and heparin (2 mg/ml; Sigma-Aldrich). After 10 days, retinoic acid (RA, 0.1 μM; Sigma-Aldrich) was added for neural caudalization. At day 17, posteriorized neuroectodermal cells were collected. These clusters were then suspended for a week in the same medium with RA (0.1 μM) and sonic hedgehog (100–200 ng/ml; R&D Systems). On day 24, BDNF (brain-derived neurotrophic factor) and GDNF (glial cell-derived neurotrophic factor) (10 ng/ml; PeproTech) were added. To enrich for motor neurons a centrifugation gradient was applied. Motor neurons were subsequently transduced with a lenti-Hb9::*GFP* construct^5^. Cells were fixed and stained for quantification using known neuronal markers (Table 1).

#### Induction of ALS-like toxicity

iPSC-derived motor neurons were exposed to ALS-like toxicity by co-culture with SOD1^G93A^ astrocytes^29^,^30^ or by glutamate overload^11^,^28^. For the ALS astrocyte toxicity assay, motor neurons were plated on the bottom chamber of a transwell co-culture system and cultured in the presence of astrocytes isolated either from SOD1^G93A^ or wild-type mice. Glial monolayers were prepared from spinal cords of newborn pups as previously described^57^. For the glutamate toxicity assay, motor neurons were cultured for 7 days in a neurotrophin-deprived medium prior to the addition of glutamate. Toxicity was induced by the addition of 1–100 μM glutamate and 100 μM L-trans-Pyrrolidine-2,4-dicarboxylic acid (PDC) for 7 days^11^. For analysis of neuroprotection, cultures were treated with recombinant IGF-2 (1–100 ng/ml, R&D Systems) either 2–4 hours prior to or 24–48 hours after induction of toxicity. Cultures were subsequently maintained for an additional 7 days for the glutamate assay and 3 weeks for the astrocyte toxicity assay.

#### SMA-like degeneration of human motor neurons

The SMA motor neurons present an apparent cell autonomous degeneration *in vitro* after 8 weeks of culturing^5^, which was used to model SMA *in vitro*. For analysis of neuroprotection cultures were treated with recombinant IGF-2 continuously (50 or 100 ng/ml) after 4 weeks of culture.

#### Immunocytochemistry of motor neuron cultures

Cells were fixed in 4% PFA for 10 min and permeabilized with 0.25% triton X-100, followed by blocking with 10% BSA in PBS and 0.3% triton X-100 for 1 hour at room temperature. Cells were incubated with primary antibodies (Table 1) overnight at 4 °C and after washing secondary antibodies were applied for 1.5 hours at room temperature (anti-goat or anti –rabbit Alexa Fluor 594 (1:400; Life Technologies). For imaging of cell cultures, a LEICA LCS2 confocal microscope was used. Controls, omitting either primary or secondary antibodies were conducted for all stainings.

#### *In vitro* quantification

Quantification of motor neuron survival *in vitro* was performed by counting 10 randomly selected fields per well. Morphometric axonal length studies were performed by measuring soma diameter and length distance to the most distal point of the axon^5^. Differences were analyzed using the Kolmogorov-Smirnov test (http://www.physics.csbsju.edu/stats/KS-test.n.plot_form.html). All quantifications were performed blind to the genetic status of the material and the treatment.

### Administration of AAV vectors to SOD1^G93A^ ALS mice, analysis of behavior, survival and quantification of effects on motor neurons and axons

A total dosage of 11 × 10^11^ particles of AAV9 vector expressing IGF-2 or GFP were injected bilaterally into the hindlimb quadriceps and intercostal muscles of SOD1^G93A^ animals at 80 days of age (n = 10 for AAV9::*IGF*-*2*, n = 5 for AAV9::*GFP*), using a Hamilton syringe. AAV9::*null* was used as a control vector (n = 15). Animals were randomized using an assigned animal identification number. Power analysis using GraphPad was performed to calculate the number of mice needed to treat to detect a difference of 10% in life-span with 80% power (β = 0.8) at a significance level of 0.05. All mice were monitored daily after AAV9::*IGF*-*2* or AAV9::*null* treatment for phenotypic hallmarks of disease. The investigators that executed the functional assessment were blind to the treatment. Body weight was recorded and motor function was tested weekly with an accelerating rotarod device (4–40 rpm; Rota-Rod 7650; Ugo Basile). The animals were sacrificed when they were unable to right themselves within 30 seconds when placed on either side^58^.

#### Motor neuron and axon counting *in vivo* after IGF-2 delivery

AAV9-injected mice were sacrificed at P140 and the lumbar region of the spinal cord was sectioned (12 μm) and Nissl stained with methylene blue^59^. The number of motor neurons in the ventral horn and soma diameter were analyzed at 40× magnification, according to previously established criteria^59^. The axonal count was performed as previously described on semi-thin transverse sections stained with toluidine blue^60^. Axon quantification was done at 60× magnification on lumbar anterior roots using a Zeiss Axiophot microscope.

#### Quantification of GAP-43 expression at the NMJ

For analysis of GAP-43 expression at the NMJ, control and IGF-2 treated mice were sacrificed when the control group reached end-stage. Muscles were dissected and immunohistochemically processed as described above. A minimum of 50 NMJs from regions across lumbrical muscles from mice treated with AAV9::*IGF*-*2* or AAV9::*null* vector were assessed. Motor endplates were individually categorized based on the level of GAP-43 expression at each one. GAP-43 levels were categorized as distinct (bright and defined staining overlying the endplate), diffuse (faint and undefined staining, or only partially overlying the endplate) or devoid (no GAP-43 overlying the endplate), Supplementary Fig. 4. All analyses and quantifications were performed blind to the genetic status of the material and the treatment.

### Statistical analysis

All statistical analyses were performed with GraphPad Prism or Stats Direct software (version 2.6.4) unless otherwise specified. When making multiple comparisons on a single data set one-way analysis of variance (ANOVA) was used and when several variables were taken into account, two-way ANOVA was used, followed by appropriate *post hoc* analysis. Two-tailed, unpaired Student’s t test was used to compare two groups. Kaplan–Meier survival analysis and the log-rank test were used for survival comparisons. The data met the assumptions of the specific statistical tests chosen, with the exception of the quantification of IGF-2 protein levels within motor neurons in human post mortem tissues, which did not display Gaussian distribution and thus was analyzed by Kruskal-Wallis in addition to ANOVA. Individual statistical tests are detailed in the figure legends; motor neuron numbers for IGF-2 and IGF receptor quantifications are listed in Supplementary Table 3. All experiments were performed in triplicate at a minimum. All results are expressed as mean ± SEM or mean ± SD.

## Additional Information

**How to cite this article**: Allodi, I. *et al*. Differential neuronal vulnerability identifies IGF-2 as a protective factor in ALS. *Sci. Rep.* **6**, 25960; doi: 10.1038/srep25960 (2016).

## Supplementary Material

Supplementary Information

## Figures and Tables

**Table 1:** Antibodies used for immunohistochemistry, immunocytochemistry and Western blot.

Target	Source	Host species	Concentration used				
			mouse tissue (central)	mouse tissue (peripheral)	human tissue	*in vitro*	Western blot
Neurofilament (165 kDa)	DSHB (2H3)	Mouse	–	1:50	–	–	–
βIII-tubulin	Millipore (MAB1637)	Mouse	–	–	–	1:200	–
CD11b	AbD Serotec (MCA711)	Rat	1:300	–	–	–	–
ChAT	Millipore (AB144P)	Goat	1:300	–	1:50	–	–
ChAT	Millipore (AB143)	Rabbit	1:200	–	–	1:200	–
Gap43	Millipore (AB5520)	Rabbit	–	1:250	–	–	–
GAPDH	Abcam (ab9485)	Rabbit	–	–	–	–	1:2500
GFAP	Dako (Z0334)	Rabbit	1:500	–	–	–	–
GSK3PSe	LifeSpan Biosciences (LS-B55)	Rabbit	–	–	–	1:200	–
pAKTSer473	Sigma (SAB4504331)	Rabbit	–	–	–	1:200	–
β-catenin	Santa Cruz (E-5)	Mouse	–	–	–	1:200	–
HB9	Millipore (ABN174)	Rabbit	–	–	–	1:200	–
IGF-2	R&D Systems (AF792)	Goat	1:40	–	1:20	–	–
IGF-2	Abcam (ab9574)	Rabbit	1:200	–	1:100	–	–
IGF-1 receptor	R&D Systems (AF-305-NA)	Goat	1:40	1:20	–	–	–
pIGF-1 receptor	Abcam (ab5681)	Rabbit	1:100	–	–	–	1:1000
pIGF-1 receptor	Abcam (ab39398)	Rabbit	–	–	–	–	1:1000
IGF-2 receptor	Abcam (ab32815)	Rabbit	1:500	1:400	–	–	1:2000
pIGF-2 receptor	Abcam (ab138453)	Rabbit	1:100	–	–	–	–
Islet1/2z	Millipore (MABN1107)	Rabbit	–	–	–	1:200	–
MAP2	Sigma (M4403)	Mouse	–	–	–	1:100	–
SMI-32	Convance (SMI-32R)	Mouse	–	–	–	1:500	–
SV2A	DSHB (SV2)	Mouse	–	1:50	–	–	–
