# Supplementary material for: Differential neuronal vulnerability identifies IGF-2 as a protective factor in ALS
Source: Sci Rep. 2016 May 16;6:25960. doi: 10.1038/srep25960 (PMC4867585; doi:10.1038/srep25960)
Supplement: Supplementary Information [file srep25960-s1.pdf]

Supplementary Information

Differential neuronal vulnerability identifies IGF-2 as a protective factor in ALS

Ilary Allodi[1,3], Laura Comley[1,3], Susanne Nichterwitz[1,3], Monica Nizzardo[2], Chiara Simone[2], Julio Aguila Benitez[1], Ming Cao[1], Stefania Corti[2,4,*] and Eva Hedlund[1,4,*]

Supplementary Table 1. Characteristics of non-demented and ALS clinical cases used for immunohistochemical analysis of IGF-2 protein level

**Case number**	**Sex**	**Age at death**	**Cause of death**	**Postmortem delay time (h:min)**	**Source**
			**Cases used for histological analysis**		
1	F	58	ND (colon cancer)	14:10	NDRI
2	F	71	ND (kidney failure)	7:10	NBB
3	M	71	ND (sepsis)	7:40	NBB
4	F	87	ND (cachexia and dehydration)	5:00	NBB
5	M	70	ND (emphysema)	4.50	NDRI
6	F	49	ALS	3:45	NBB
7	M	65	ALS	10:30	NDRI
8	M	71	ALS	6:45	NBB
9	M	74	ALS	7:20	NDRI
10	M	62	ALS	6:55	NBB

NBB - Netherland's Brain Bank (http://www.brainbank.nl)
NDRI - National Disease Research Interchange (http://www.ndriresource.org/)

Supplementary Table 2. Characteristics of human fibroblast-derived induced pluripotent stem cell (iPSC) lines

iPSC line	Diagnosis	Sex	Age	Reprogramming strategy	Reference
27b	ALS (SOD1/G85S)	Female	29	Retrovirus, 3 factors: OSK	[1]
29d	ALS (SOD1/L144F)	Female	82	Retrovirus 4 factors: OSKM	[1]
AL/ALS 1.1	ALS (SOD1/L144F)	Female	55	Non viral 6 factors: OSKM+LN	This report
SC603A/B-ALS	ALS, sporadic	Female	61	Retrovirus, 4 factors: OSKM	www.systembio.com
AM/ALS 1.1	ALS, sporadic	Female	77	Non viral 6 factors: OSKM+LN	This report
iPS Foreskin	Healthy Donor	Male	newborn	Lentivirus, 4 factors: OSNL	[2]
19.9	Healthy Donor	Male	newborn	Non viral 6 factors: OSKM+LN	[3]
CP13c	Healthy Donor	Female	45	Non viral 6 factors: OSKM+LN	This report
11b	Healthy Donor	Male	36	Retrovirus 3 factors: OSK	[1]
15b	Healthy Donor	Female	48	Retrovirus 3 factors: OSK	[1]
**iPSC line**	**Diagnosis**	**Sex**	**Age**	**Reprogramming strategy**	**Reference**
SMA 1.1	SMA I	Male	3	Non viral 6 factors: OSKM+LN	[4]
SMA 2.1	SMA I	Male	2	Non viral 6 factors: OSKM+LN	[4]
19.9	Healthy Donor	Male	newborn	Non viral 6 factors: OSKM+LN	[3]
CP13c	Healthy Donor	Female	45	Non viral 6 factors: OSKM+LN	This report

References

1. Boulting, G.L., *et al.* A functionally characterized test set of human induced pluripotent stem cells. *Nat Biotechnol* **29**, 279-286 (2011).
2. Yu, J., *et al.* Induced pluripotent stem cell lines derived from human somatic cells. *Science* **318**, 1917-1920 (2007).
3. Yu, J., *et al.* Human induced pluripotent stem cells free of vector and transgene sequences. *Science* **324**, 797-801 (2009).
4. Corti, S., *et al.* Genetic correction of human induced pluripotent stem cells from patients with spinal muscular atrophy. *Sci Transl Med* **4**, 165ra162 (2012).

Supplementary Table 3. Number of motor neurons used to quantify IGF-2 and IGF-1R signal intensity in immunostained tissues

	**Tissue origin**	**Motor neuron counts**		
		**CNIII**	**CNXII**	**SC**
**IGF-2**	Wild-type mice, P126	58	71	59
	SOD1^{G93A} mice, P126	69	82	93
	ND Control patients	140	123	86
	ALS patients	222	171	170
**pIGF-1R**	Wild-type mice, P126	54	-	61
	SOD1^{G93A} mice, P126	76	-	67
**IGF-1R**	Wild-type mice, P126	42	-	31
	SOD1^{G93A} mice, P126	48	-	22
**pIGF-2R**	Wild-type mice, P126	57	-	37
	SOD1^{G93A} mice, P126	67	-	37

Supplementary Figure 1. Analysis of IGF-2 and IGF receptors in wild-type and SOD1^{G93A} mice. Protein quantification was assessed by immunohistochemistry. Statistical analysis utilizing 2-way ANOVA with "genotype" and "motor neuron group" as factors was performed for IGF-2 levels and showed a main effect of "motor neuron group" ($F(2,426)=97.03$, $P<0.0001$), and the interaction between "genotype" and "MN group" ($F(2,426)=8.08$, $P=0.0004$). *Post hoc* analysis showed that the interaction was driven by a significant difference in spinal motor neurons between wild-type and SOD^{G93A} animals at the P126 time point ($P=0.0202$) (**a,** n=3 per genotype). Expression of phosphorylated IGF-1R did not differ in oculomotor and spinal motor neurons between wild-type and SOD^{G93A} animals (**b,** n=3 per genotype, 2-way ANOVA), however a main effect of "motor neuron group" was detected ($F(1,254)=91.18$, $P<0.0001$) reinforcing the data shown in figure 2e. Overall levels of IGF-1R were assessed with a pan marker (**c-f**). 2-way ANOVA showed a main effect of "motor neuron group" ($F(1,139)=46.17$, $P<0.0001$), and *post hoc* analysis revealed significantly higher IGF-1R levels in oculomotor neurons compared to spinal motor neurons for both genotypes ($P<0.0001$) (**g**). Immunofluorescent staining and confocal imaging showed that (**h**) pIGF-1R protein was present at very low levels in spinal motor neurons of SOD^{G93A} mice and more prominently in (**i**) glial cells surrounding the motor neurons in the SOD^{G93A} mice. (**j**) Extracts from extraocular muscles from wild-type and SOD^{G93A} animals were run on the same gel and after transfer membranes were cut for staining with different antibodies. Both antibodies, directed either against the Y1161 or against the Y1158, Y1162, and Y1163 phosphorylation sites of the IGF-1R protein showed bands of around 130 kDa in size. Phosphorylated IGF-2R levels analyzed by immunohistochemistry were compared with 2-way ANOVA. A main effect of "genotype" was detected ($F(1,194)=7.10$, $P=0.0083$) and *post hoc* analysis showed a significant difference between wild-type and SOD^{G93A} animals in oculomotor neurons ($P=0.041$). Scale bar: f: 20 μM (applicable to c-e) h: 50 μM, i: 20 μM.

Supplementary Figure 2. IGF-1R staining on extraocular muscles co-localized with acetylcholine receptor staining of motor endplates. Immunofluorescent staining of (**a-c**) extraocular (EOM) muscles and (**d-f**) lumbrical muscles with bungarotoxin (BTX; 488 nm, green) and an anti-IGF-1R antibody (647 nm, blue), showed that IGF-1R expression co-localized with AChR staining in EOMs (**a, c**), while the staining was below detection level in lumbricals (**d, f**). High magnification confocal images (and orthogonal views) showed that IGF-1R was mainly co-localized with the postsynaptic motor endplates (**g-i**), although some IGF-1R was expressed in the incoming motor axon (**g**). Scale bar: f: 20 μm (applicable to a-f), i: 20 μm (applicable to g-i).

Supplementary Figure 3. IGF-2 added prior to SOD1 astrocyte or glutamate-induced toxicity protects human spinal motor neurons in culture. (**a**) Schematic drawing (by Mattias Karlen) of the transwell co-culture system of motor neurons and muscle. iPSC derived motor neurons were vulnerable to (**b**) co-culture with mutant $SOD1^{G93A}$ astrocytes and to (**c**) increased levels of glutamate. In the presence of 20μM glutamate, a dose dependent effect of IGF-2 on motor neuron survival could be seen (**d**). sALS and fALS motor neurons were more sensitive to both (**e**) $SOD1^{G93A}$ astrocytes ($F(2,87)=18.28$, $P<0.001$, ANOVA) and (**f**) glutamate ($F(2,87)=36.69$; $P<0.001$, ANOVA) toxicity respect to wild-type cells (n=10 independent experiments in triplicate; $P<0.001$, ANOVA). Treatment of cultures with IGF-2 (50 ng/ml) 2-4 hours prior to induction of toxicity by either co-culture with (**g**) mutant $SOD1^{G93A}$ astrocytes or (**h**) glutamate insult protected motor neurons (SOD1 astrocytes, $P<0.0001$, ANOVA. Glutamate, $P<0.0001$, ANOVA; n= 5 independent experiments in triplicate per condition). Values represent means ± SD. Confocal micrographs of cultures (**g**) after 3 weeks of combined IGF-2 and mutant SOD1 astrocyte co-culture or (**h**) 7 days of combined IGF-2 and glutamate treatment show large numbers of motor neurons (visualized by their expression of Hb9-GFP). Scale bar =50μm in d.

Supplementary Figure 4. Quantification of GAP-43 expression at the NMJ. Levels of GAP-43 staining varied between individual NMJs. NMJs were therefore subdivided into three different categories for the purposes of quantification, based on the level of GAP-43 expression. (**a**) Expression was considered to be distinct when there was bright GAP-43 immunoreactivity, with a defined structure juxtaposing the motor endplate. (**b**) NMJs with faint or undefined GAP-43 staining and those where the GAP-43 staining did not cover the majority of the endplate (>75%) were considered to have diffuse staining. (**c**) Motor endplates with no corresponding GAP-43 staining were considered to be devoid of GAP-43. Scale bar = 20μm.

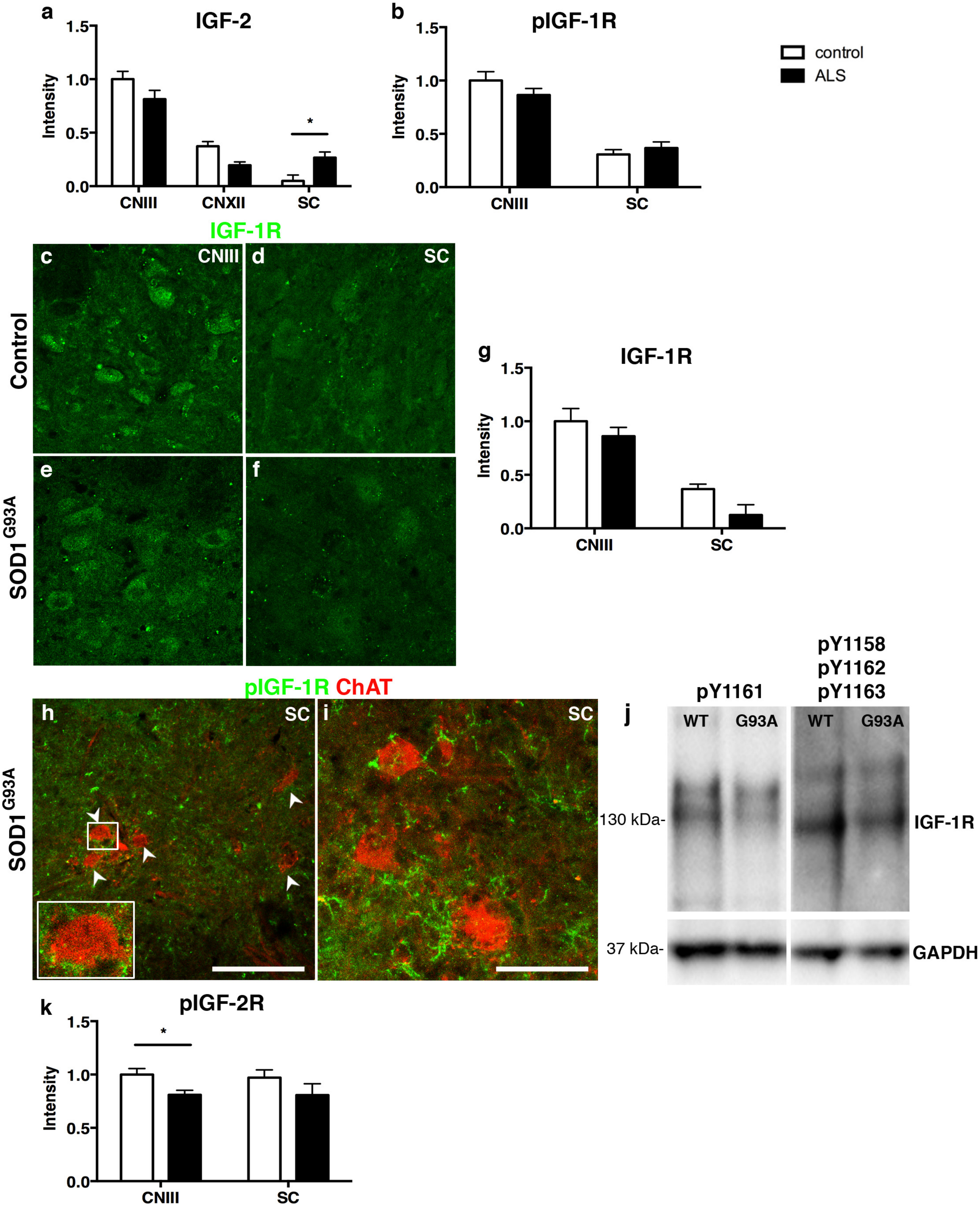
a
IGF-2
Intensity
1.5
1.0
0.5
0.0
*
CNIII
CNXII
SC
b
pIGF-1R
Intensity
1.5
1.0
0.5
0.0
CNIII
SC
control
ALS
IGF-1R
c
CNIII
d
SC
Control
e
f
SOD1G93A
g
IGF-1R
Intensity
1.5
1.0
0.5
0.0
CNIII
SC
pIGF-1R ChAT
h
SC
i
SC
SOD1G93A
j
pY1161
pY1158
pY1162
pY1163
WT
G93A
WT
G93A
130 kDa-
IGF-1R
37 kDa-
GAPDH
k
pIGF-2R
Intensity
1.5
1.0
0.5
0.0
*
CNIII
SC

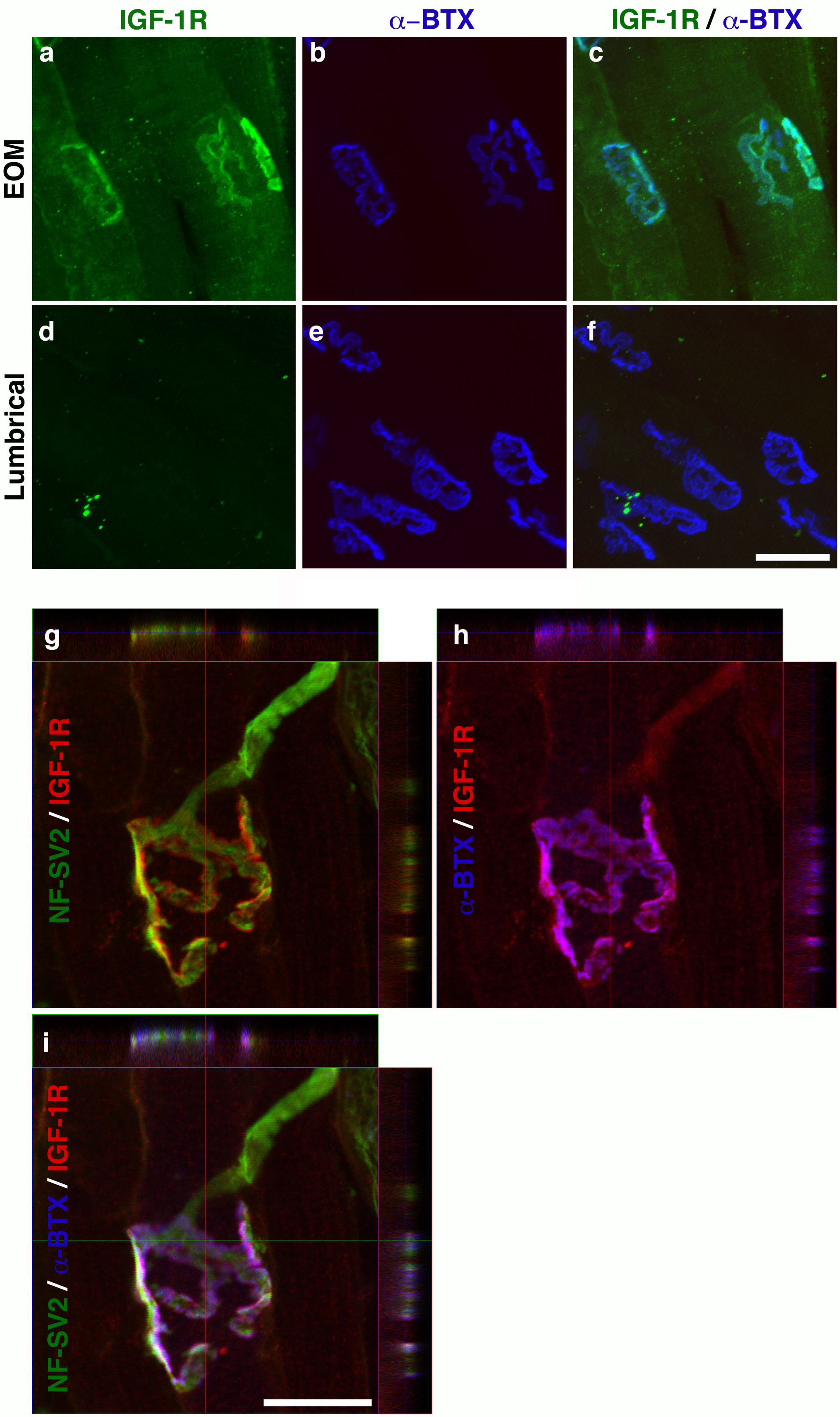

IGF-1R
α–BTX
IGF-1R / α-BTX
a
b
c
EOM
d
e
f
Lumbrical
g
NF-SV2 / IGF-1R
h
α-BTX / IGF-1R
i
NF-SV2 / α-BTX / IGF-1R

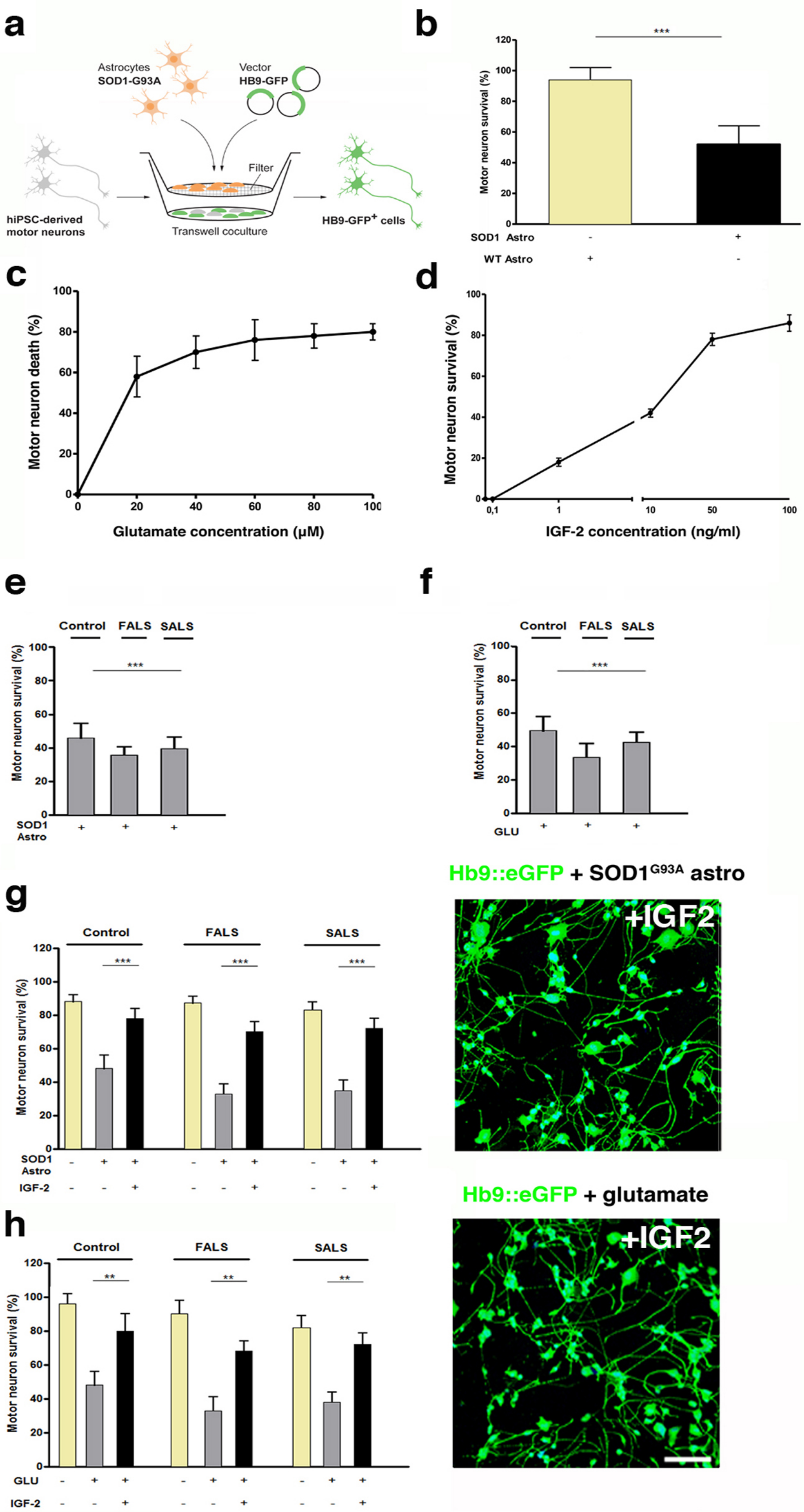
a
Astrocytes
SOD1-G93A
Vector
HB9-GFP
Filter
hiPSC-derived
motor neurons
Transwell coculture
HB9-GFP+ cells
b
Motor neuron survival (%)
SOD1 Astro
WT Astro
c
Motor neuron death (%)
Glutamate concentration (μM)
d
Motor neuron survival (%)
IGF-2 concentration (ng/ml)
e
Control
FALS
SALS
Motor neuron survival (%)
SOD1
Astro
f
Control
FALS
SALS
Motor neuron survival (%)
GLU
g
Control
FALS
SALS
Motor neuron survival (%)
SOD1
Astro
IGF-2
Hb9::eGFP + SOD1G93A astro
+IGF2
h
Control
FALS
SALS
Motor neuron survival (%)
GLU
IGF-2
Hb9::eGFP + glutamate
+IGF2

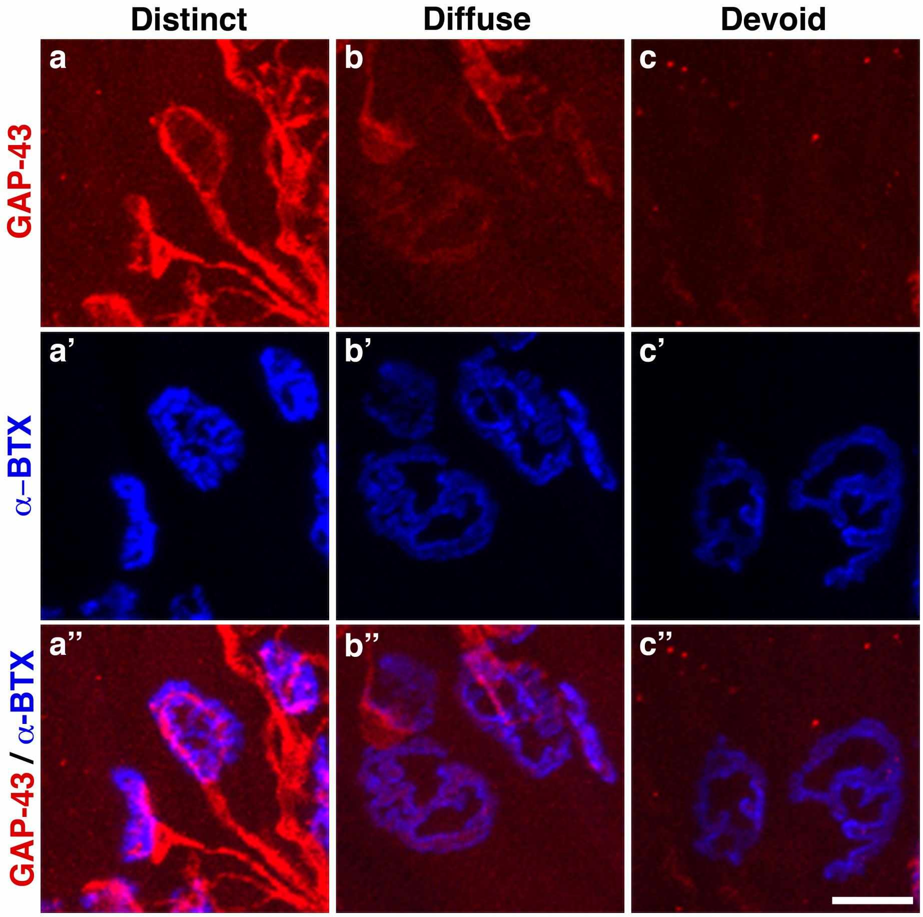
Distinct
Diffuse
Devoid
GAP-43
α–BTX
GAP-43 / α-BTX
a
b
c
a’
b’
c’
a’’
b’’
c’’
